# Inhibiting Amadori-modified albumin formation improves biomarkers of podocyte damage in diabetic rats

**DOI:** 10.1002/phy2.83

**Published:** 2013-09-17

**Authors:** Margo P Cohen, Clyde W Shearman

**Affiliations:** GlycadiaPhiladelphia, Pennsylvania

**Keywords:** Amadori-modified albumin, nephrin, podocalyxin, podocyte damage, βig-h3 protein

## Abstract

Recent studies have shown that urinary excretion of podocyte proteins is an indicator of podocyte injury, and that podocyte abnormalities and elevated concentrations of Amadori-modified glycated albumin (AGA) are linked to the development of diabetic nephropathy and to each other. We evaluated relationships between urinary markers of podocyte damage, increased AGA and filtration function in rats made diabetic by streptozotocin injection and treated for 8 weeks with a compound that inhibits the formation of AGA, with age-matched nondiabetic and diabetic rats serving as controls. Blood and urine were collected for measurement of glycated albumin, creatinine, albumin, nephrin, podocalyxin, and βig-h3 protein. The elevated circulating concentrations of glycated albumin and higher urinary levels of these podocyte markers as well as of albumin that were observed in diabetic rats compared with nondiabetic controls were significantly reduced in animals receiving test compound, and decrease in urinary biomarkers correlated with reduction in AGA. The results provide evidence that lowering the concentration of AGA, independent of filtration status and hyperglycemia, reduces urinary nephrin, podocalyxin, and βig-h3 protein, linking the increased glycated albumin associated with diabetes to podocyte abnormalities and shedding of podocyte proteins into the urine.

## Introduction

Although the pathogenesis of diabetic nephropathy remains incompletely delineated, results of recent studies support the hypothesis that damage to glomerular podocytes and abnormalities in podocyte function are intimately involved in the development of diabetic renal disease and in the increased albumin excretion that heralds its onset (Ellis et al. 1987; Parving et al. 1992, 2002; Pagtalunan et al. 1997; Altonen et al. 2001; Steffes et al. 2001; Langham et al. 2002; Toyoda et al. 2004). Injury to glomerular epithelial cells gives rise to defacement and shedding, with loss of the podocyte protein nephrin that regulates the passage of plasma proteins into Bowman's space and disruption of the integrity of the glomerular filtration barrier. Increased urinary nephrin, which has been observed in both experimental and human diabetes, is paradoxical to a decrease in nephrin mRNA, correlates positively with the degree of proteinuria, and may be a useful marker of diabetic renal dysfunction (Luimula et al. 2000; Forbes et al. 2002; Langham et al. 2002; Patari et al. 2003; Toyoda et al. 2004; Chang et al. 2012; Jim et al. 2012). Other urinary analytes of relevance in assessing podocyte and glomerular damage include podocalyxin, the 140 kDa main podocyte sialoglycoprotein that coats the cell surface and provides a protective negative charge that limits passage of negatively charged plasma proteins through the filtration barrier (Kerjaschki et al. 1984), and the ∼68 kDa beta-inducible-gene protein (βig-h3) that is induced by transforming growth factor (TGF)-β, secreted into the extracellular matrix, and involved in cell growth, differentiation, adhesion, and wound healing (Skonier et al. 1992, 1994; LeBaron et al. 1995). Notwithstanding that podocalyxin is not specific to the podocyte, urinary podocalyxin has been found to be a useful marker of disease activity and podocyte shedding in various disorders of the kidney (Kanno et al. 2003; Peterman and Floege 2007; Wang et al. 2012). The dependence of βig-h3 on TGF-β, coupled with recognition of the important pathogenetic role of TGF-β in diabetic nephropathy and in podocyte function (Yamamoto et al. 1993; Sharma and Ziyadeh 1995; Reeves and Andreoli 2000; Ziyadeh et al. 2000; Wolf et al. 2005), prompted examination of the urinary concentration of βig-h3 as a means of assessing biological activity of renal TGF-β1 and renal dysfunction in diabetes, with the report that levels were increased in the urine of patients with type 2 diabetes, without or with increased urine albumin excretion, compared to control subjects and showed a positive correlation with albumin excretion (Ha et al. 2004; Cha et al. 2005).

A link between podocyte abnormalities and elevated levels of Amadori-modified glycated albumin (AGA) associated with diabetes was established with the demonstration that lowering the circulating concentration of AGA without change in blood glucose levels restores the reduced nephrin that is observed in the kidneys of diabetic mice (Cohen et al. 2005, 2007). AGA has been shown to influence cell signaling and molecular mediators participation in the pathogenesis of diabetic nephropathy including upregulation of the TGF-β1 and vascular endothelial growth factor (VEGF) systems, stimulation of the production of matrix proteins, and activation of protein kinase (PK)C-β1 and extracellular signal-regulated kinase (ERK) (Cohen and Ziyadeh 1994; Ziyadeh et al. 1998; Cohen et al. 1999; Chen et al. 2001; Cohen et al. 2001). The relevance of such findings to the development of renal dysfunction in vivo was corroborated by the demonstration that decreasing the elevated AGA levels in diabetic mice significantly reduces the overexpression of TGF-β1 and VEGF, and ameliorates matrix accumulation, glomerular histomorphometric changes, and the development of renal insufficiency (Cohen et al. 2002, 2005, 2007). Reduction in AGA independent of any effect on hyperglycemia was achieved by administration of a small molecule designated 23CPPA that interacts noncovalently with albumin binding pockets and impedes the formation of glucose adducts at potentially glycatable lysine amino groups in the albumin protein (Cohen et al. 2002, 2005, 2007).

The foregoing considerations concerning AGA as a causally contributory factor in podocyte dysfunction and increased urinary levels of nephrin, podocalyxin, and βig-h3 as a reflection of podocyte damage prompted authors of this study to evaluate the effect of 23CPPA on these analytes. These experiments employed the streptozotocin diabetic rat, a model of insulin-dependent diabetes that exhibits hyperfiltration of early onset and long duration (O'Donnell et al. 1988; Stackhouse et al. 1990), thereby affording the opportunity to explore relationships of these analytes with filtration function as well as with each other and with AGA. The experimental protocol elected a time course consistent with that employed by others in this model in which alterations in glomerular histology, matrix proteins, TGF-β1 expression, smad2/3 and ERK1/2 signaling, and urinary TGF-β1 have been documented (D'Agord Schaan et al. 2001; Chen et al. 2011; Quilley et al. 2011). We report that lowering plasma AGA by administration of 23CPPA reduces the elevated urinary excretion of these analytes as well as of albumin, which is increased in the streptozotocin diabetic rat, but not the hyperfiltration that is observed in this experimental animal model of diabetes.

## Materials and Methods

### Experimental animals and treatment protocol

Male Wistar rats (Harlan; Indianapolis, IN) aged 6 weeks and weighing between 120 and 140 g were made diabetic by intravenous injection (50 mg/kg) of streptozotocin (Sigma-Aldrich, St. Louis, MO) into the tail vein. All diabetic animals had blood glucose levels, measured using a One Touch Glucometer (Lifescan, Milpitas, CA), greater than 250 mg/dL at 48 h after streptozotocin injection. Age-, weight-, and gender-matched Wistar rats (*n* = 8) served as nondiabetic controls. The diabetic rats were divided into two groups, one of which (*n* = 10) served as the diabetic control and the other (*n* = 11) received the test compound as the potassium salt. The drug was administered by gavage at a dose of 10 mg kg^−1^ day^−1^ in two equally divided portions for eight consecutive weeks commencing 3 days after induction of diabetes. This dosage corresponded to that employed in previous studies in diabetic rodents in which 23CPPA has been shown to reduce glycated albumin concentrations without affecting hyperglycemia (Cohen et al. 2002, 2005, 2007). All diabetic rats received long-acting insulin (Lantos; Aventis, Bridgewater, NJ) every other day with adjustments of dosage to prevent ketoacidosis and to keep the animals on a positive growth curve. Commercial rodent chow and water were provided ad libitum. The rats were housed in a temperature-controlled facility and the institutional Animal Care and Use Committee approved all procedures. Urine samples were collected for 24 h in metabolic cages, and blood was sampled via the tail vein; after measurement of urine volume and separation of plasma by centrifugation, specimens were stored frozen at −80°C until assay.

### Analytic methods

Urinary concentrations of albumin, nephrin, podocalyxin and βig-h3 were determined by immunospecific assay, and urine creatinine was measured using a colorimetric procedure employing picric acid, all according to the manufacturer's instructions (Exocell, Philadelphia, PA). The albumin and nephrin assays are competitive immunoassays with specificities for the rat proteins with respective sensitivities to 0.1 and 0.02 μg/mL, and have intra- and interassay coefficients of variation <5%. The podocalyxin assay is a competitive enzyme-linked immunoassay (ELISA) immunospecific for the rat protein with a sensitivity to 0.5 ng/mL and intra- and interassay coefficients of variation of <7%. The βig-h3 assay is a sandwich ELISA immunospecific for the rodent protein with a sensitivity to 10 pg/mL and intra- and interassay coefficients of variation of <7%. For measurement of plasma creatinine, the method was modified by including a deproteination step with acid tungstate, and the alkaline picric reagent was added after absorption with Fuller's earth, which eliminates interference by confounding chromogens that may falsely elevate plasma creatinine (Dunn et al. 2004). Plasma glycated albumin was determined as nmol hydroxymethylfurfuraldehyde (Ney et al. 1981) per nmol albumin (BCA; Pierce, Rockford, IL) after absorption of albumin to Cibacron Blue Agarose 3GA (Sigma-Aldrich, St. Louis, MO) and elution with 2.5 mol/L NaCl (Cohen and Hud 1989).

### Statistical analysis

Data are presented as means ± SEM. Differences among groups were determined by analysis of variance (ANOVA), and correlations were calculated by Pearson's correlation analysis. A *P*-value of <0.05 was considered statistically significant.

## Results

### Experimental animal characteristics

General characteristics of the animals conformed to those typically found in this model of experimental diabetes (Table 1). Body weights 8 weeks after protocol initiation were lower in both groups of diabetic rats compared to the nondiabetic controls. Both groups of diabetic animals exhibited marked polyuria and blood glucose concentrations were elevated to a similar extent in diabetic rats whether or not receiving test compound, corroborating, as previously demonstrated, that the compound does not affect glycemic status (Cohen et al. 2002, 2005, 2007, 2008). The growth curves and blood glucose levels in the animals in this study were similar to those reported by others using streptozotocin diabetic rats receiving insulin without or with coadministration of an agent that does not affect hyperglycemia (Thallas-Bonke et al. 2008). Plasma concentrations of glycated albumin were elevated in diabetic compared to nondiabetic control rats, and were significantly lower in diabetic rats receiving 23CPPA compared to the diabetic controls, indicating that the test compound impeded nonenzymatic glycation of circulating albumin despite exposure to a hyperglycemic milieu, as has been previously reported (Cohen et al. 2002, 2005, 2007). The mean plasma creatinine concentration was lower in diabetic controls and diabetic-treated compared to nondiabetic control rats but did not reach statistical significance (*P* = 0.08 and *P* = 0.06). Creatinine clearance values were approximately 50% higher in diabetic compared to nondiabetic controls, and were not significantly different from diabetic controls in diabetic rats receiving 23CPPA.

**Table 1:** Animal characteristics

	Nondiabetic	Diabetic control	Diabetic treated
Body weight (g)	467 ± 12	297 ± 8*	312 ± 12*
Blood glucose (mmol/L)	7.6 ± 1.2	26.1 ± 1.9*	25.5 ± 1.0*
Urine volume (mL/24 h)	7.7 ± 0.8	145 ± 17*	150 ± 18*
Glycated albumin (nmHMF/nm albumin)	1.1 ± 0.01	1.45 ± 0.15*	1.23 ± 0.06**
Plasma creatinine (mg/dL)	0.36 ± 0.02	0.31 ± 0.02	0.30 ± 0.02
Creatinine clearance (mL/min)	1.74 ± 0.16	2.53 ± 0.20*	2.96 ± 0.44*

* *P* < 0.05 compared to nondiabetic control.

** *P* < 0.05 compared to diabetic control.

### Biomarker analysis

**Figure 1:**
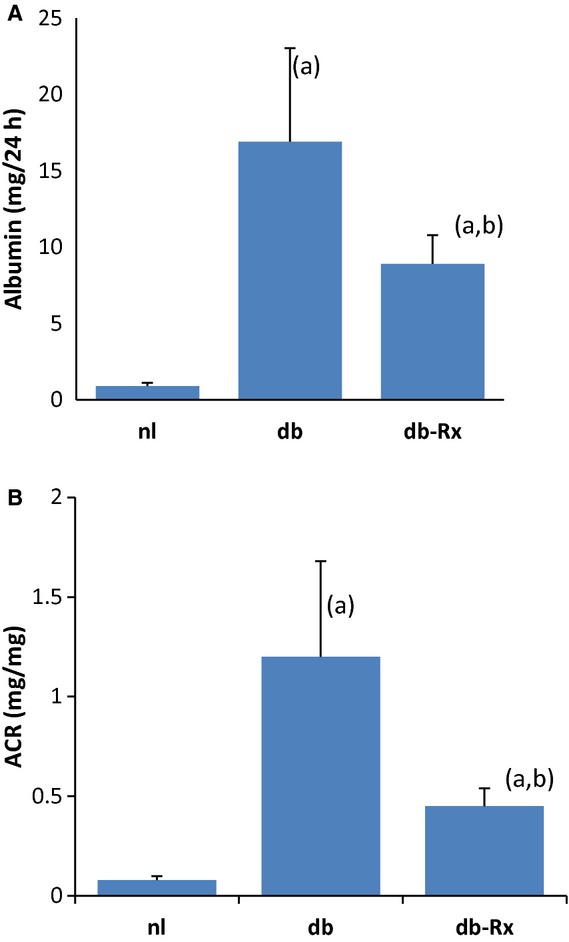
Urine albumin excretion. Urine albumin in nondiabetic control (nl), diabetic control (db), and 23CPPA-treated diabetic (db-Rx) rats measured as mg albumin/24 h (A) or mg albumin/mg creatinine (B). Data for A and B are mean ± SEM. (a) *P* < 0.05 compared to nondiabetic control. (b) *P* < 0.05 compared to diabetic control.

Urine albumin excretion was markedly increased in diabetic compared to nondiabetic animals and was significantly reduced in diabetic rats receiving 23CPPA compared to the diabetic controls, with values ∼50% less than those of diabetic controls whether expressed as mg/24 h (Fig. 1A) or as mg/mg creatinine (Fig. 1B). This decrease in albumin excretion was due to the treatment regimen and could not be ascribed to relative caloric deprivation as body weights in the diabetic controls were not significantly different from those in diabetic rats that received test compound. There was a significant positive linear correlation (*r* = 0.98) between the 24-h urinary albumin excretion and the albumin:creatinine ratio (ACR) for all animals. The incomplete normalization of albumin excretion in the diabetic rats receiving test compound is consistent with the persistent and marked hyperglycemia in these animals.

**Figure 2:**
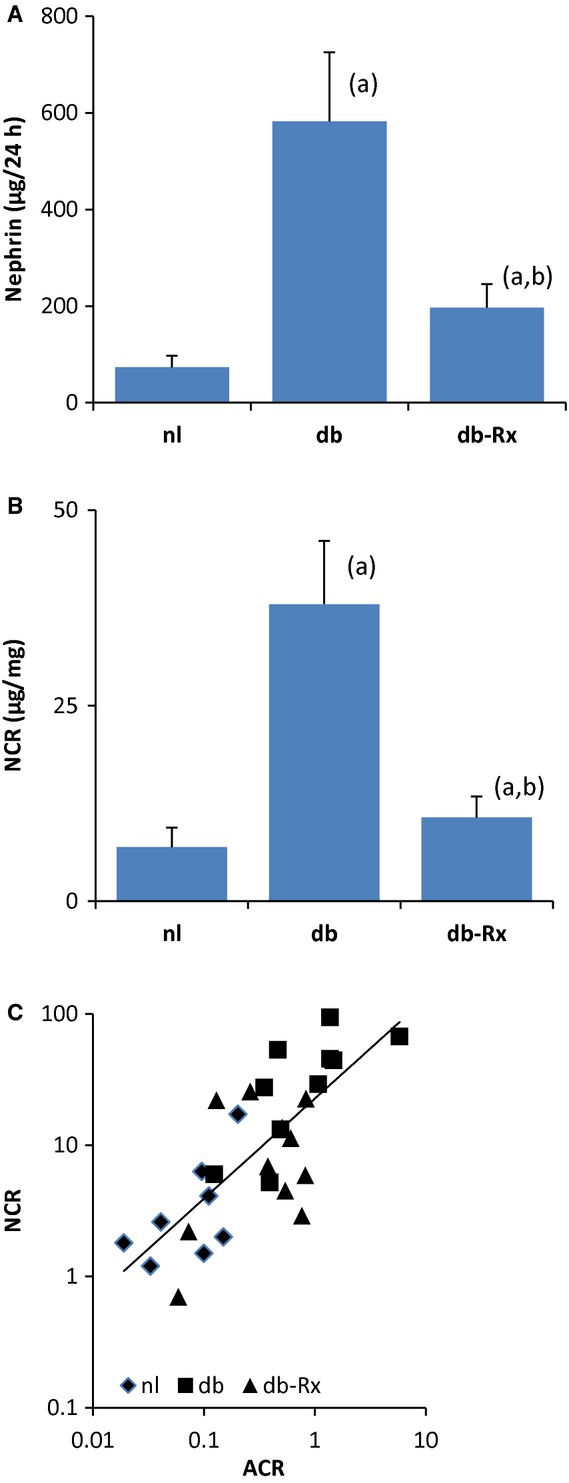
Urine nephrin excretion. Urine nephrin in nondiabetic control (nl), diabetic control (db), and 23CPPA-treated diabetic (db-Rx) rats, measured as μg nephrin/24 h (A) or μg nephrin/mg creatinine (B). There was a significant positive linear correlation (*r* = 0.63) between the ACR and NCR in all animals (C). Data for A and B are mean ± SEM. (a) *P* < 0.05 compared to nondiabetic control. (b) *P* < 0.05 compared to diabetic control.

As shown in Figure 2, urine nephrin was increased in diabetic compared to nondiabetic controls, with mean values that were significantly higher when expressed as either μg/24 h (Fig. 2A) or as μg/mg creatinine (Fig. 2B). Administration of test compound to diabetic rats significantly reduced urine nephrin, with values ∼65–70% less than those of the diabetic controls whether expressed as μg/24 h (Figure 2A) or as μg/mg creatinine (Fig. 2B). There was a significant positive linear correlation (*r* = 0.94) between the 24-h urine nephrin excretion and the nephrin:creatinine ratio (NCR) for all animals. There was also a significant positive linear correlation (*r* = 0.63) between the NCR and ACR (Fig. 2C), consistent with the hypothesis that podocyte dysfunction participates in the genesis of albuminuria. Figure 2C also depicts the relationship between urine albumin and urine nephrin concentrations in each of the three experimental groups, illustrating parallel reduction in both analytes in diabetic rats that received the test compound.

**Figure 3:**
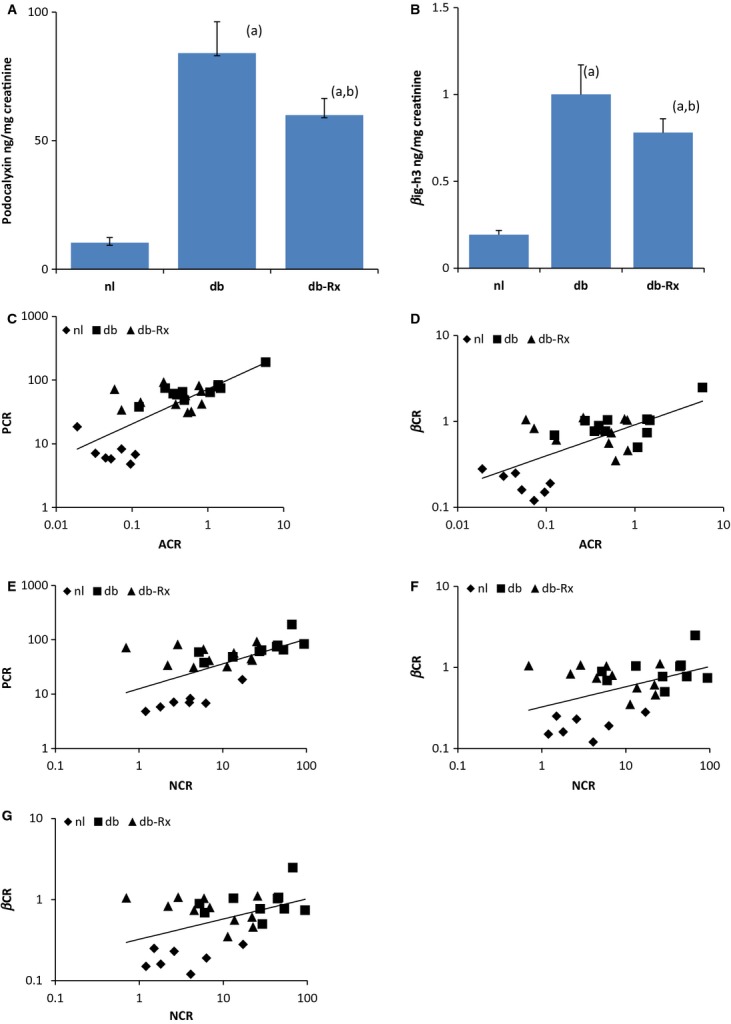
Urine podocalyxin and βig-h3 excretion. Urinary podocalyxin and βig-h3 in nondiabetic control (nl), diabetic control (db), and 23CPPA-treated diabetic (db-Rx) rats, measured as ng podocalyxin/mg creatinine (PCR; A) and ng βig-h3/mg creatinine (βCR; B). There were significant positive linear correlations between the ACR and PCR (*r* = 0.82; C); between the ACR and βCR (*r* = 0.77; D); between the NCR and PCR (*r* = 0.64; E); between the NCR and βCR (*r* = 0.45; F); and between the PCR and βCR (*r* = 0.93; G). Data for A and B are mean ± SEM. (a) *P* < 0.05 compared to nondiabetic control. (b) *P* < 0.05 compared to diabetic control.

Figure 3 presents urine levels of podocalyxin and βig-h3, normalized to urine creatinine (PCR and βCR), in the three groups of experimental animals. Both analytes were markedly elevated in diabetic compared to the nondiabetic control animals, and both analytes were significantly less in samples from diabetic rats treated with test compound compared to the diabetic controls, with respective values ∼30% and 20% less than those of the diabetic controls (Fig. 3A and B). There were significant positive correlations between the PCR and the ACR (*r* = 0.82) and between the βCR and the ACR (*r* = 0.77) (Fig. 3C and D). The PCR and βCR also showed significant positive correlations with the NCR (*r* = 0.64 and *r* = 0.45, respectively), and with each other (*r* = 0.93) as shown in Figure 3E–G.

**Figure 4:**
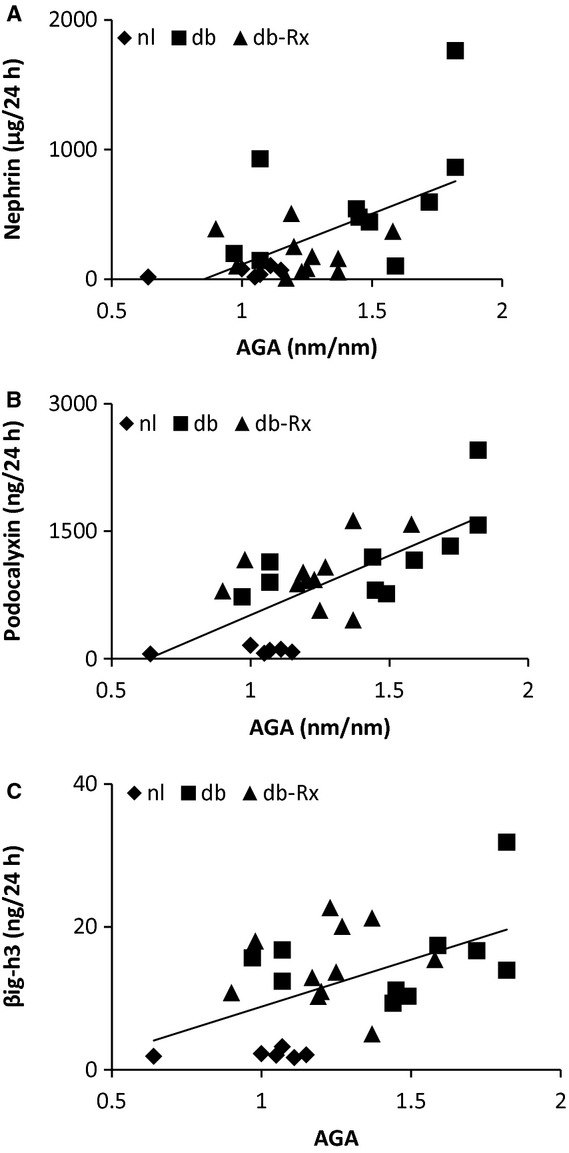
Plasma AGA and urinary biomarkers. Plasma AGA, measured as nmHMF/nm albumin, in relation to urinary analytes in nondiabetic control (nl), diabetic control (db), and 23CPPA-treated diabetic (db-Rx) rats. There were significant positive linear correlations between AGA and urine nephrin (*r* = 0.58; A), podocalyxin (*r* = 0.70; B), and βig-h3 (*r* = 0.50; C).

Figure 4 presents plasma levels of AGA in relation to urinary biomarkers of podocyte damage, illustrating parallel reduction in these analytes and AGA in diabetic rats that received the test compound. There were significant positive linear correlations between AGA and urine nephrin (*r* = 0.58; Fig. 4A), podocalyxin (*r* = 0.70; Fig. 4B), and βig-h3 (*r* = 0.50, Fig. 4C).

## Discussion

This study employed ELISA methodology to quantify urinary excretion of nephrin, podocalyxin, and βig-h3 in rats with streptozotocin diabetes to explore relationships of these analytes with each other and with albumin excretion, and to determine if administration of a compound that inhibits the formation of AGA affects these markers of podocyte and glomerular dysfunction. The experimental protocol, initiated at ∼6 weeks of age and continued for eight consecutive weeks, was consistent with that employed in other studies using the streptozotocin diabetic rat to assess abnormalities in early diabetic nephropathy. For example, glomerular expression of fibronectin, collagen IV and TGF-β1,smad2/3, and ERK1/2 signaling, urinary TGF-β1 and renal glucose transporter (GLUT 1) have been found to be increased after 6–8 weeks of diabetes, and these parameters are improved with various interventions of 2–3 months duration (D'Agord Schaan et al. 2001; Chen et al. 2011; Quilley et al. 2011). The present results demonstrate that urinary excretion of nephrin, podocalyxin, and βig-h3 are elevated in this rodent model of insulin-deficient diabetes and that the urinary levels of these analytes show significant positive correlations with each other and with albumin excretion, supporting the hypothesis that nephrinuria and podocalyxinuria reflect the presence and severity of dysfunction of the glomerular filtration barrier. The results also indicate that reducing the concentration of AGA in diabetes significantly decreases urinary nephrin and podocalyxin excretion, supporting the hypothesis that elevated concentrations of AGA in diabetes contribute to podocyte dysfunction. Increased urinary nephrin is paradoxical to the decreased glomerular nephrin expression that has been shown in mouse models of diabetic nephropathy and that is restored with 23CPPA (Cohen et al. 2005, 2007; Chang et al. 2012). Thus, reducing AGA ameliorates both compromised nephrin production and podocyte shedding.

The observation that urinary βig-h3 also decreased in animals receiving the inhibitor of AGA formation, albeit more modestly than did urinary albumin, nephrin or podocalyxin, is consistent with the stimulation of TGF-β expression that is induced by AGA and with the attenuation in the overexpression of glomerular TGF-β that accompanies reduction in AGA concentrations in genetically diabetic rodents treated with this compound (Ziyadeh et al. 1998; Chen et al. 2001; Cohen et al. 2002). Although not considered a major factor in the development of albuminuria, βig-h3 is associated with the extracellular matrix where it serves an adhesive function through interaction with alpha3 beta1 integrin (Bae et al. 2002; Jeong and Kim 2004; Park et al. 2004). TGF-β1 promotes morphologic changes in and apoptosis of podocytes, and reduces glomerular basement membrane adhesion of podocytes through downregulation of alpha3 beta1 expression (Ziyadeh and Wolf 2008; Dessapt et al. 2009; Herman-Edelstein et al. 2011). Urinary βig-h3 may thus reflect podocyte damage and sloughing in the context of TGF-β1 mediated disruption of βig-h3 and alpha3 beta1 adhesion.

The modest decrease in plasma creatinine concentrations in diabetic compared to nondiabetic rats is compatible with the increase in creatinine clearance observed in these animals. Creatinine clearance may be considered an imperfect method for measuring the glomerular filtration rate (GFR) but, because this study employed the same methodology in all animals, the higher creatinine clearance values in diabetic compared to nondiabetic rats after 8 weeks of diabetes are viewed as corresponding with the hyperfiltration of early onset and long duration that has been observed in this model of insulin deficient diabetes (O'Donnell et al. 1988; Stackhouse et al. 1990), which may not be accompanied by a significant change in plasma creatinine (Benigni et al. 2003). The creatinine clearance values in nondiabetic animals reported in this study approximate the inulin (Wesslau et al. 1988) and creatinine clearance (Luft et al. 1976; Stackhouse et al. 1990) values reported in other studies. Factors contributory to an increased GFR in diabetes, in addition to hyperglycemia per se, include elevated capillary pressure, alterations in various vasoactive mediators such as increased prostaglandin production, and decreased sensitivity of the tubuloglomerular feedback mechanism (Luft et al. 1976; Wesslau et al. 1988; Stackhouse et al. 1990). Given that AGA does not influence creatinine clearance, it is not surprising that lowering AGA levels with 23CPPA did not affect hyperfiltration in diabetic rats, which reflects the marked and persistent hyperglycemia in both diabetic control and treated rats and was not significantly different in these experimental groups. The role of glomerular hyperfiltration as a risk factor for renal outcome in diabetes is not clear (O'Donnell et al. 1988; Anderson and Vora 1995; Yip et al. 1996; Pitrosch et al. 2005; Sallstrom et al. 2007), but the lessening of urinary nephrin, podocalyxin, and βig-h3 as well as albumin without change in creatinine clearance in animals receiving test compound indicates that these decreases do not reflect effects on the glomerular filtration rate and that hyperfiltration is not a principal contributor to the increases in control diabetic animals.

In summary, we provide evidence that, 8 weeks after the induction of diabetes, streptozotocin-diabetic rats manifest increased urine levels of nephrin, podocalyxin, and βig-h3 that show positive correlations with each other as well as with albuminuria, and that administration of a compound that lowers the concentration of AGA significantly decreases urinary levels of these analytes, linking the elevated concentrations of AGA associated with diabetes to the abnormalities resulting in podocyte dysfunction and shedding of podocyte proteins in the urine.
